# Pediatric pyopneumothorax caused by *Prevotella oris* successfully diagnosed via mNGS: a case report and literature review

**DOI:** 10.3389/fmed.2026.1888298

**Published:** 2026-07-23

**Authors:** Zhihui Yan, Xiaona Qian, Yixuan Liu, Liyuan Tian

**Affiliations:** 1 Department of Respiratory Medicine, Children’s Hospital of Hebei Province, Shijiazhuang, Hebei, China; 2 Hebei Provincial Clinical Research Center for Child Health and Disease, Shijiazhuang, Hebei, China

**Keywords:** anaerobic infection, case report, empyema, metagenomic next-generation sequencing (mNGS), *Prevotella oris*, pyopneumothorax

## Abstract

**Background:**

Empyema and pyopneumothorax are severe complications of pediatric community-acquired pneumonia. While typically caused by aerobic bacteria, anaerobic infections, particularly those involving *Prevotella oris* (*P. oris*), are exceedingly rare in children. This study aims to explore the clinical characteristics, diagnostic challenges, and therapeutic strategies for pediatric pyopneumothorax caused by *P. oris*, thereby enhancing clinical awareness of this uncommon opportunistic pathogen.

**Case presentation:**

We retrospectively analyzed the clinical data of a 10-year-old male admitted to the Hebei Children’s Hospital in October 2025, presenting with acute chest pain and a history of tooth extraction 1 week prior to symptom onset. Radiological imaging revealed bilateral pneumonia with bilateral pleural effusions (predominantly on the left side). Pleural fluid analysis was consistent with an empyema. Traditional bacterial cultures of blood and pleural fluid yielded negative results. However, probe-based targeted metagenomic next-generation sequencing (mNGS) of the pleural fluid identified *P. oris* with a high relative abundance (81.86%), alongside other minor oral commensals. Based on the molecular diagnosis and the patient’s ongoing clinical deterioration, cefoperazone-sulbactam was selected to strengthen coverage against anaerobic Gram-negative organisms, while linezolid was temporarily added to cover potential Gram-positive pleural co-infection during the acute deterioration phase. This was combined with closed thoracic drainage and intrapleural urokinase instillation for fibrinolysis, leading to a complete clinical recovery.

**Conclusion:**

*Prevotella oris* is a rare but significant pathogen in pediatric empyema. A high index of suspicion should be maintained for anaerobic infections in children presenting with a history of dental procedures, abnormal immune parameters or possible immunological vulnerability, or poor response to empirical antibiotics. Traditional cultures are often inadequate; therefore, mNGS serves as a crucial tool for the early detection and precise treatment of difficult-to-culture anaerobes.

## 1 Introduction

Empyema, characterized by the accumulation of purulent fluid in the pleural space, is a critical complication of pediatric community-acquired pneumonia, occurring in approximately 2% of cases (1). The predominant pathogens in the pediatric population are typically aerobic bacteria, such as *Streptococcus pneumoniae* and *Staphylococcus aureus*. In contrast, anaerobic bacteria are relatively uncommon culprits, generally associated with poor oral hygiene, aspiration events, or an immunocompromised state (2, 3).

*Prevotella oris* (*P. oris*) is an obligate anaerobic, Gram-negative bacillus that constitutes a normal part of the human oral, respiratory, and gastrointestinal microbiome (4). As an opportunistic pathogen, *P. oris* can breach mucosal barriers during dental procedures or in the setting of immunodeficiency, leading to endogenous infections through hematogenous spread or contiguous extension (5). While adult cases of *Prevotella*-induced lung abscesses and empyema have been sporadically documented, pediatric pyopneumothorax caused specifically by *P. oris* is exceptionally rare, and literature is largely confined to isolated case reports.

The clinical diagnosis of anaerobic empyema remains highly challenging. Traditional culture methods exhibit low sensitivity and demand stringent transport and incubation conditions, frequently resulting in false-negative outcomes and subsequent delays in targeted therapy (6). In recent years, metagenomic next-generation sequencing (mNGS) has increasingly improved the etiological diagnosis of infectious diseases (7). By bypassing the need for culture, mNGS offers a rapid, unbiased, and highly sensitive approach to identifying fastidious and rare pathogens, including anaerobes like *Prevotella* spp. (8, 9).

**Figure 1:**
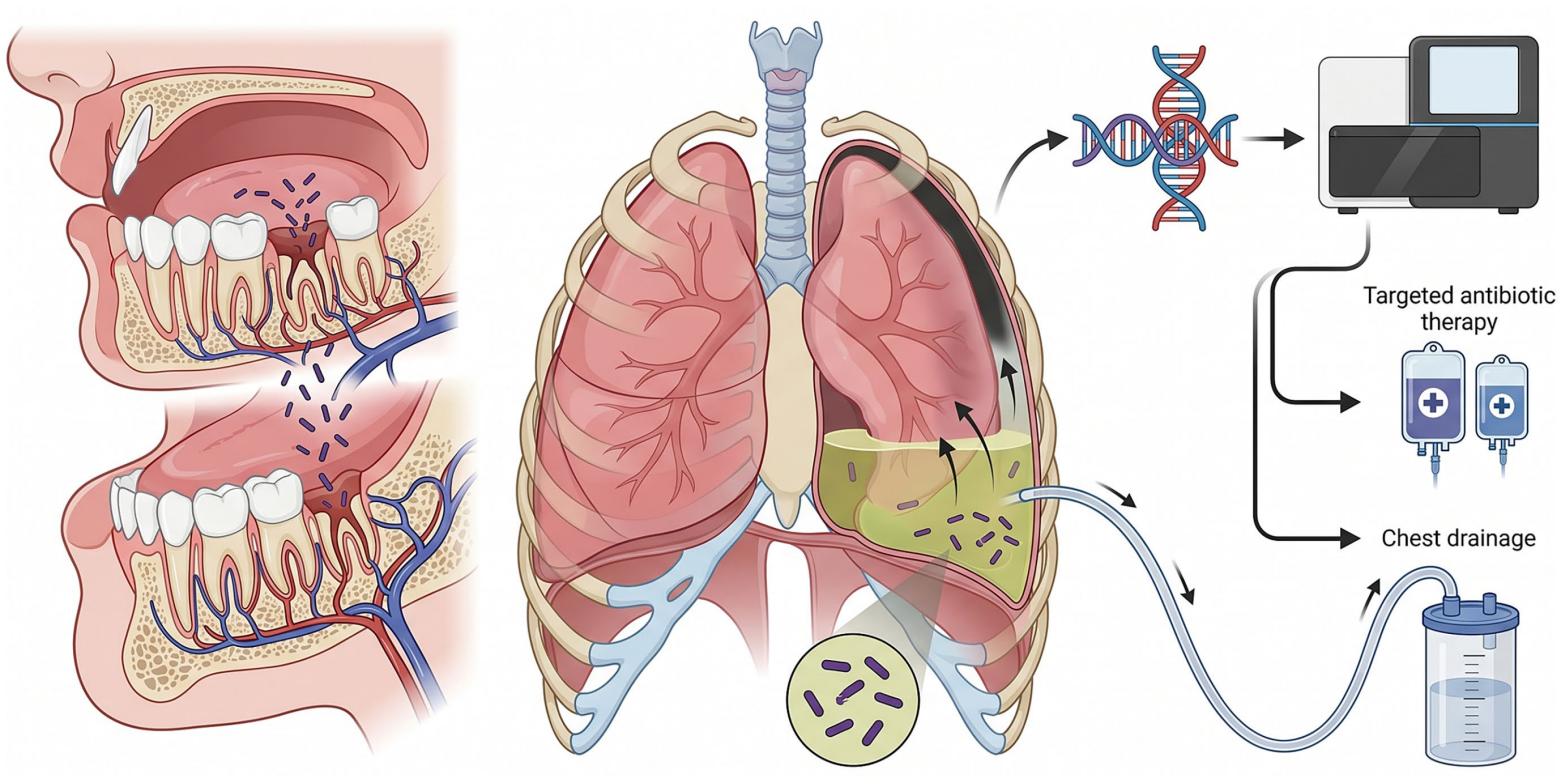
Clinical diagnostic workflow. A schematic summary of the suspected odontogenic origin, pleural infection, molecular diagnosis, and targeted management of pediatric pyopneumothorax caused by *Prevotella oris*. Following dental extraction, oral microbiota including *P. oris* may translocate to the pleural cavity, resulting in severe left-sided pyopneumothorax. Probe-based metagenomic next-generation sequencing of pleural effusion enabled pathogen identification when conventional cultures were negative, facilitating timely antimicrobial adjustment and source-control treatment.

In this article, we report a rare case of a 10-year-old boy who developed a severe pyopneumothorax following a dental extraction, which was ultimately diagnosed as a *P. oris* infection via mNGS after empirical treatments and conventional cultures failed. To enhance the robustness of this report and adhere to international academic standards, this manuscript was prepared following the CARE (CAse REports) guidelines (10). Furthermore, we provide a comprehensive literature review to elucidate the clinical features, diagnostic modalities, and optimal management of this rare entity. A schematic clinical diagnostic workflow is illustrated in Figure 1.

## 2 Case presentation

### 2.1 Patient information and initial clinical findings

A 10-year-old male child was admitted to our hospital on October 20, 2025, presenting with severe left-sided chest pain that had persisted for 3 days and rapidly exacerbated over the preceding 12 h. The patient had no antecedent typical respiratory symptoms such as fever or cough. Upon admission, his body temperature was normal (36.2 °C). Further inquiry into his medical history revealed that the patient had undergone a tooth extraction 1 week prior to the onset of the chest pain.

**Figure 2:**
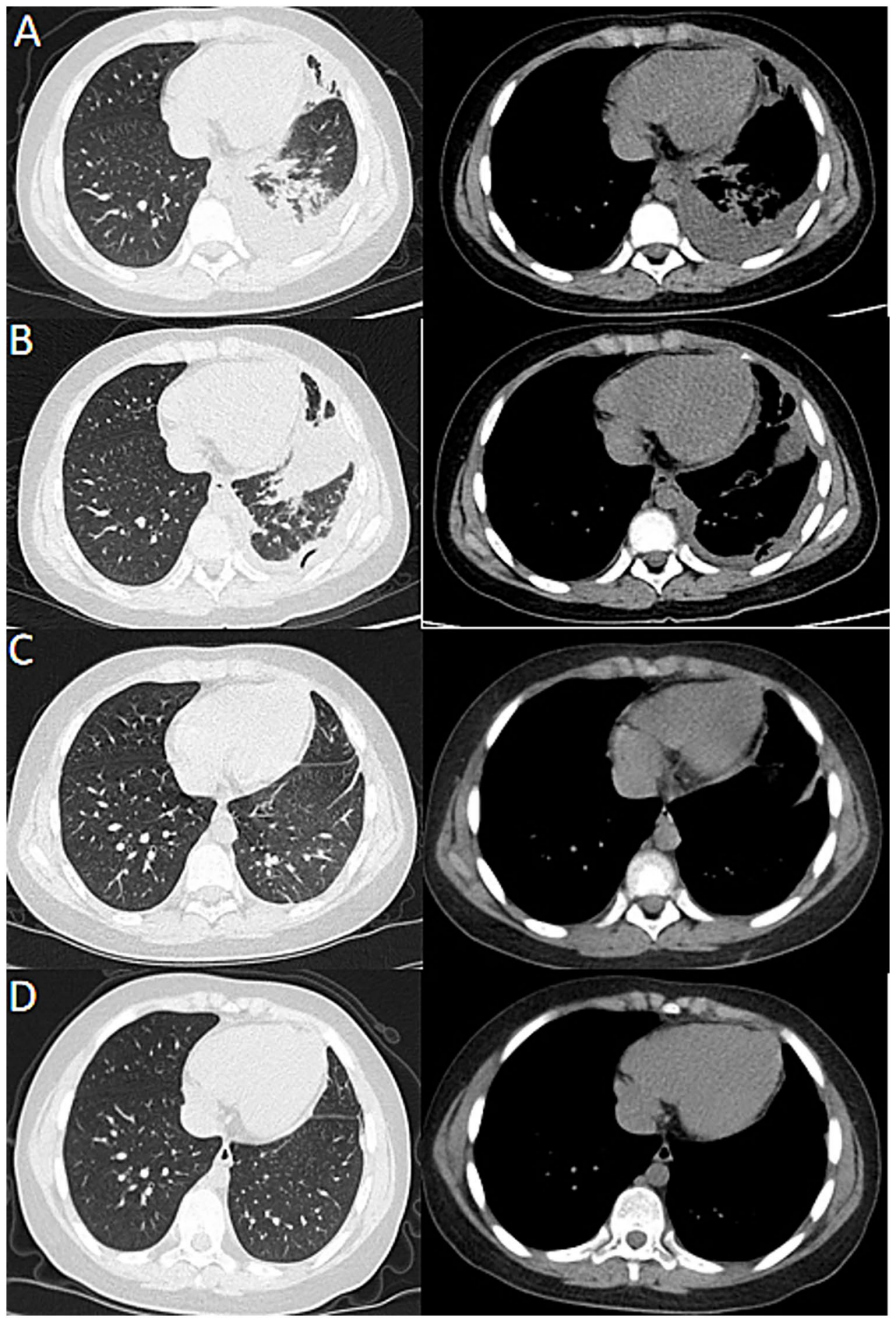
Representative chest computed tomography (CT) scans during the clinical course. **(A)** Initial CT on admission (Oct 20, 2025) demonstrating bilateral pulmonary infiltrates with severe left-sided pyopneumothorax and pleural effusion. **(B)** Follow-up CT on Oct 27, 2025, showing partial absorption of the pulmonary inflammation and reduced pleural effusion following closed thoracic drainage and intrapleural urokinase administration. **(C)** CT on Nov 4, 2025 (prior to discharge), indicating marked improvement and near-complete resolution of the effusion. **(D)** Long-term follow-up CT on December 19, 2025, confirming complete resolution without significant residual sequelae.

Initial laboratory investigations indicated a significant inflammatory response: the white blood cell (WBC) count was 17.53 × 10^9^/L with a neutrophil predominance (87.5%), C-reactive protein (CRP) was elevated at 26.80 mg/L, and interleukin-6 (IL-6) surged to 493.30 pg./mL. A computed tomography (CT) scan of the chest upon admission revealed bilateral pneumonia accompanied by bilateral pleural effusions, which were markedly more severe on the left side (Figure 2A).

### 2.2 Empirical treatment and immunological assessment

Based on the initial findings, empirical antimicrobial therapy was promptly initiated with intravenous cefotaxime sodium/sulbactam sodium, supplemented by ambroxol for expectoration and beclomethasone dipropionate for anti-inflammatory purposes. On the following day (October 21), a closed thoracic drainage was performed. The drained fluid was yellowish, slightly turbid, and purulent. Routine analysis of the pleural fluid confirmed the diagnosis of empyema, showing a total nucleated cell count of 19,304 × 10^6^/L, of which 95.7% were polymorphonuclear leukocytes.

A 13-panel multiplex PCR for respiratory pathogens returned positive only for Rhinovirus. Additionally, absolute enumeration of lymphocyte subsets revealed a decline in cellular and humoral immunity: helper T cells (Th) were at 40.46%, suppressor T cells (Ts) at 16.27% (Th/Ts ratio: 2.49), and double-negative T cells at 8.36%. Absolute counts were consistently low (total T cells: 324/μL, Th: 199/μL, Ts: 80/μL, total B cells: 79/μL, Natural Killer cells: 86/μL), suggesting transient lymphopenia and possible immunological vulnerability during the acute infection.

### 2.3 Pathogen identification via mNGS and targeted treatment

Despite the empirical therapy, the patient’s condition deteriorated, evidenced by increased febrile episodes. By October 23, the WBC count remained high at 13.21 × 10^9^/L (neutrophils 75.7%), and CRP drastically increased to 135.87 mg/L. Traditional bacterial cultures of both blood and pleural fluid yielded negative results after 48 h. Because obligate anaerobes and oral microbiota may require strict anaerobic transport and prolonged incubation for up to approximately 1 week, the 48-h negative culture result was interpreted cautiously and did not exclude anaerobic infection.

To identify the elusive pathogen, the pleural fluid was subjected to target-enrichment high-throughput metagenomic next-generation sequencing (MetaCAP™, Tianjin Kingmed Center for Clinical Laboratory). The mNGS results identified *Prevotella oris* as the predominant organism. Specifically, the sequencing yielded 4,386 specific read sequences for *P. oris*, corresponding to a remarkably high relative abundance of 81.86% and a genome coverage of 2.55%. Other oral microbiota detected included *Parvimonas micra* (331 reads, 5.82%), *Dialister pneumosintes* (79 reads, 1.39%), *Campylobacter rectus* (55 reads, 1.01%), and *Tannerella forsythia* (15 reads, 0.26%), as well as Torque teno virus (9 reads). Given the patient’s recent history of tooth extraction, the overwhelmingly high read count and relative abundance of *P. oris* strongly pointed to an odontogenic translocation leading to pleural infection. The other minimally detected oral commensals were clinically interpreted as minor co-commensals or transient background microbial signals rather than primary drivers of the severe empyema.

Upon receiving the mNGS report on October 23, and given the patient’s ongoing clinical deterioration, cefoperazone sodium/sulbactam sodium was selected to strengthen coverage against anaerobic Gram-negative organisms, while linezolid was temporarily added to cover potential Gram-positive pleural co-infection during the acute deterioration phase.

### 2.4 Comprehensive management and clinical outcome

Due to poor drainage caused by highly viscous and loculated purulent fluid, intrapleural instillation of urokinase was initiated on October 24 to dissolve the fibrinous septations. This intervention successfully increased the drainage volume. During the disease course, the patient developed transient hepatic dysfunction (ALT 65 U/L, GGT 119 U/L) and a significant elevation in D-dimer (11.47 mg/L). These complications were effectively managed with hepatoprotective agents and subcutaneous heparin sodium for anticoagulation.

Following this comprehensive and targeted management strategy, the patient’s condition improved remarkably. By October 27, the WBC count dropped to 8.12 × 10^9^/L, CRP decreased to 44.93 mg/L, and procalcitonin (PCT) was 0.89 μg/L. A follow-up CT scan demonstrated partial absorption of the pulmonary infiltrates and a significant reduction in the pleural effusion (Figure 2B). By November 2, CRP normalized, and D-dimer decreased to 6.67 mg/L. A subsequent CT on November 4 showed continued marked improvement (Figure 2C).

**Figure 3:**
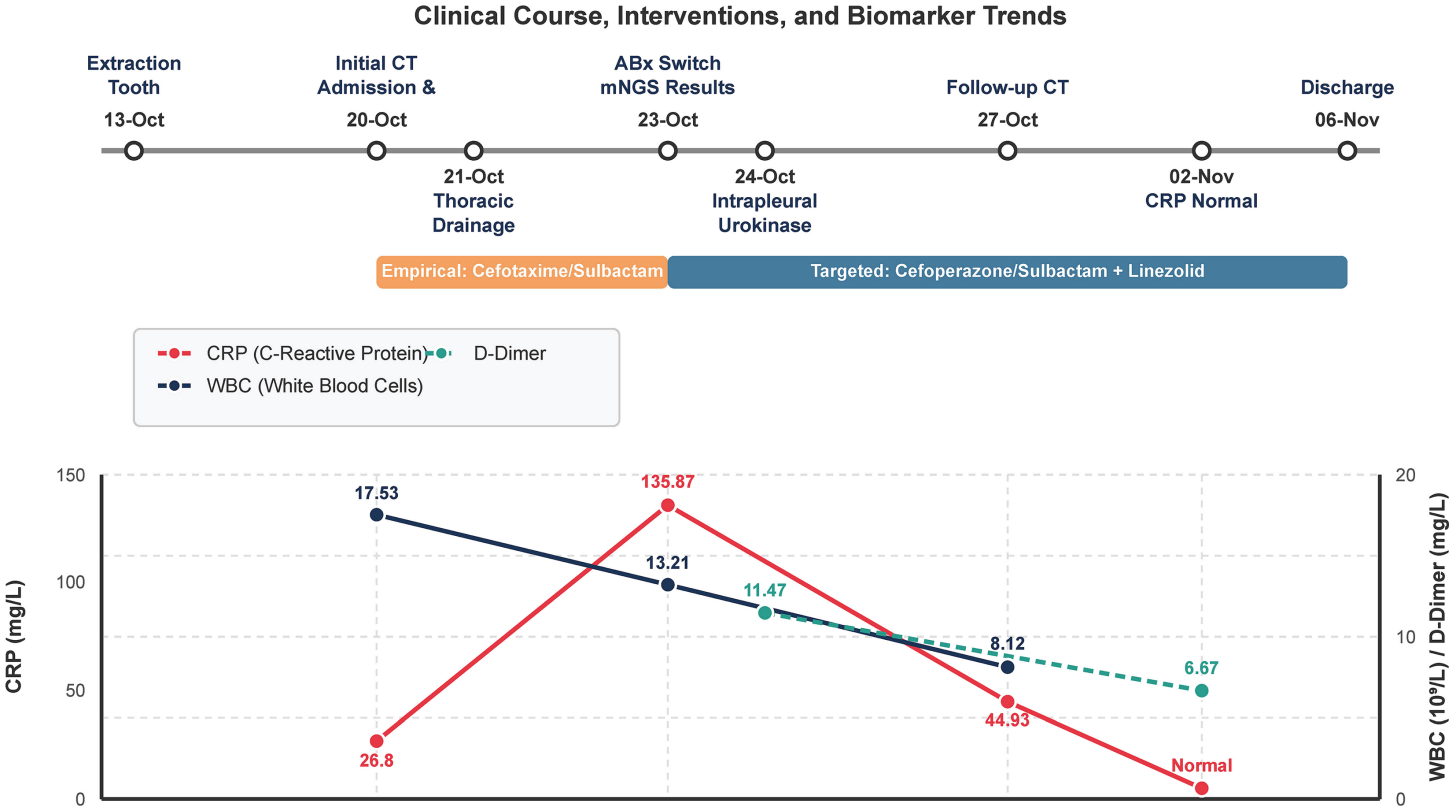
Clinical timeline of the patient. A schematic timeline demonstrating the progression of symptoms (starting from tooth extraction), major clinical interventions (hospital admission, thoracic drainage, antibiotic switch, and intrapleural urokinase application), and the corresponding dynamic trends of key inflammatory biomarkers including White Blood Cell (WBC) count, C-Reactive Protein (CRP), and D-dimer levels until hospital discharge.

The patient achieved clinical resolution and was discharged on November 6. Upon discharge, oral linezolid and cefcapene pivoxil were prescribed for a 10-day consolidation therapy. A long-term follow-up CT performed on December 19 confirmed the complete resolution of the pneumonia and pyopneumothorax without residual sequelae (Figure 2D). A comprehensive timeline depicting the clinical course, symptom onset, therapeutic interventions, and inflammatory biomarker trends is presented in Figure 3.

## 3 Discussion

*Prevotella oris* is a Gram-negative anaerobic bacillus belonging to the genus *Prevotella*. It is a well-known commensal of the human mucosal surfaces, particularly the oral cavity, gastrointestinal tract, and urogenital tract (11, 12). Although generally considered an opportunistic pathogen, *P. oris* can cause severe endogenous infections when mucosal barriers are disrupted or host immunity is compromised (13). While infections typically manifest in the oral cavity, female genital tract, or abdominal cavity, pleuropulmonary infections—especially pyopneumothorax in pediatric patients—are exceedingly rare (14). To our knowledge, this is the first reported case in China of pediatric pyopneumothorax caused by *P. oris* that was definitively diagnosed via mNGS.

The pathogenesis of *P. oris* in pleuropulmonary infections is strongly associated with the aspiration of oropharyngeal secretions or hematogenous dissemination following oral procedures (15, 16). In our case, the 10-year-old patient presented with acute chest pain but lacked typical prodromal respiratory symptoms like coughing. Notably, he had a history of tooth extraction 1 week prior to admission. Furthermore, his immunological profile revealed a decreased absolute count of T and B lymphocytes, suggesting possible host immunological vulnerability during the acute infectious episode. We hypothesize that the dental extraction compromised the oral mucosal barrier, allowing the commensal *P. oris* to enter the bloodstream and seed the pleural cavity. This hematogenous route, possibly facilitated by transient cellular immune vulnerability, led to a rapidly progressive empyema and subsequent pyopneumothorax (17, 18).

Diagnosing anaerobic empyema using conventional microbiological methods is notoriously difficult. Traditional cultures often fail due to the fastidious nature of anaerobes, prior antibiotic exposure, or inadequate anaerobic transport systems (19). In this case, both blood and pleural fluid cultures were negative. The application of target-enrichment mNGS (MetaCAP™) provided a crucial breakthrough. The sequencing detected a remarkably high read count (4,386) and relative abundance (81.86%) of *P. oris* in the pleural fluid. While other oral microbiota, such as *Parvimonas micra* and *Campylobacter rectus*, were also detected, their relatively minuscule abundances (mostly <5%) identified them as either minor co-pathogens or transient background microbial signals secondary to the primary *P. oris* infection (20, 21). This precise molecular detection allowed for the swift transition from an ineffective empirical therapy to targeted antimicrobial management. We acknowledge that a 48-h culture period is insufficient to fully exclude slow-growing anaerobic bacteria and oral microbiota; therefore, the negative cultures should be regarded as a limitation of conventional testing in this patient rather than definitive evidence of absent viable anaerobes.

To further contextualize this rare presentation, we conducted a comprehensive literature review of *Prevotella*-associated severe infections, summarizing the clinical characteristics, diagnostic approaches, and outcomes in Supplementary Table 1. As demonstrated, most reported cases occur in adults with significant comorbidities or predisposing factors, such as dental disease, diabetes, or immunosuppression (22–28). Pediatric cases are extremely sparse and predominantly involve spinal or central nervous system infections rather than empyema (29, 30). Crucially, our review highlights a paradigm shift in diagnostic modalities: recent successful diagnoses overwhelmingly rely on mNGS following negative traditional cultures (31, 32).

Therapeutically, *Prevotella* species generally exhibit high susceptibility to metronidazole, carbapenems, and β-lactam/β-lactamase inhibitor combinations. However, emerging data from European multicenter studies show rising resistance rates against penicillin, clindamycin, and even moxifloxacin (33, 34). In our patient, initial therapy with cefotaxime/sulbactam was suboptimal. Guided by the mNGS findings and the patient’s ongoing clinical deterioration, cefoperazone/sulbactam was selected to strengthen coverage against anaerobic Gram-negative organisms, while linezolid was temporarily added to cover potential Gram-positive pleural co-infection during the acute deterioration phase, followed by clinical improvement and a rapid decline in WBC count and CRP. This clinical course supports a low threshold for adding or strengthening anaerobic coverage in pediatric empyema when there is a compatible odontogenic or aspiration-related history, culture negativity, or persistent clinical deterioration despite standard empirical therapy.

Beyond targeted antibiotics, effective source control is paramount in managing complicated empyema. Fibrinous septations in the pleural space often impede proper drainage, leading to clinical failure. In complicated empyema with viscous or loculated pleural collections, intrapleural urokinase may lyse loculations, improve drainage, and reduce the need for more invasive surgical interventions like video-assisted thoracoscopic surgery (VATS) (35).

## 4 Conclusion

*Prevotella oris* is a rare but highly virulent pathogen capable of causing life-threatening pyopneumothorax in pediatric patients, particularly following dental procedures. This case underscores the necessity of maintaining a high index of suspicion for fastidious anaerobic infections when empirical treatments fail and conventional cultures remain negative. Target-enrichment mNGS serves as an invaluable diagnostic tool, offering rapid and precise pathogen identification. Ultimately, a multidisciplinary approach combining mNGS-guided targeted antibiotics, timely closed thoracic drainage, and intrapleural fibrinolytic therapy is crucial for achieving optimal clinical outcomes without the need for surgical decortication.

## Data Availability

The original contributions presented in the study are included in the article/Supplementary material, further inquiries can be directed to the corresponding author.
