# Supplementary material for: Pediatric pyopneumothorax caused by Prevotella oris successfully diagnosed via mNGS: a case report and literature review
Source: Front Med (Lausanne). 2026 Jul 23;13:1888298. doi: 10.3389/fmed.2026.1888298 (PMC13446130; doi:10.3389/fmed.2026.1888298)
Supplement: Supplementary file 1 [file Table_1.DOCX]

**Supplementary Table 1. Summary of key reported cases and studies of severe infections caused by Prevotella species in the literature.**

| **Ref.** | **Author (Year)** | **Infection Site/Type** | **Major Risk Factors / Underlying Diseases** | **Diagnostic Method** | **Main Antimicrobial Therapy** | **Surgery** | **Outcome** |
| --- | --- | --- | --- | --- | --- | --- | --- |
| [22] | Zeng et al. (2021) | Lung abscess | Periodontitis, poor oral hygiene | mNGS | Moxifloxacin | No | Good |
| [23] | Mukhopadhyay (2005) | Vertebral osteomyelitis | Recent dental cleaning | Biopsy & Culture | Piperacillin/Tazobactam | Yes | Good |
| [24] | Goyal et al. (2012) | Epidural abscess | EGD procedure, esophageal cyst | Blood & Fluid Culture | Ampicillin/Sulbactam + Metronidazole | No | Good (Cured) |
| [25] | Opoku-Darko (2017) | Spondylodiscitis | Long-term steroid use | Biopsy & Culture | Piperacillin/Tazobactam | Yes | Good |
| [26] | Mo et al. (2018) | Intracranial mixed infection | Alcoholism, alcoholic liver disease | mNGS & Culture | Ceftriaxone (poor efficacy alone) | No | Poor (Withdrew) |
| [27] | Li et al. (2022) | Lung abscess | Poor oral hygiene, Diabetes, COPD | mNGS | Imipenem + Metronidazole | No | Good |
| [28] | Zhang et al. (2022) | Lung abscess | Newly diagnosed diabetes | mNGS (BALF + Blood) | Ornidazole + Clindamycin | No | Good |
| [29] | Karatay et al. (2015) | Intradural abscess (Child) | UTI, congenital spinal anomaly | Surgical Culture | Vancomycin + Metronidazole | Yes | Good |
| [30] | Tan et al. (2017) | Bacteremia | Abdominal infection, advanced age | Blood Culture | Carbapenems / Metronidazole | No | 25% Mortality |
| [31] | Zhou et al. (2024) | Bloodstream infection | Surgery, tumors, diabetes, catheters | Blood Culture | Carbapenems / Metronidazole | No | 78.3% Good |
| [32] | Wang et al. (2024) | Spinal infection | No obvious underlying disease | mNGS (Tissue) | Ceftriaxone + Metronidazole | Yes | Good |
| [33] | Brook et al. (1993) | Osteomyelitis/Arthritis | Vascular disease, trauma, bites | Culture | Clindamycin, Metronidazole | Yes | 81% Cured |
| [34] | Veloo et al. (2020) | Multi-site (Multicenter) | Antibiotic exposure variability | Etest/EUCAST | Varied (Metronidazole preferred) | N/A | N/A |
